# How Valid and Responsive Are Generic Health Status Measures, such as EQ-5D and SF-36, in Schizophrenia? A Systematic Review

**DOI:** 10.1016/j.jval.2011.04.006

**Published:** 2011-09

**Authors:** Diana Papaioannou, John Brazier, Glenys Parry

**Affiliations:** 1 School of Health and Related Research, University of Sheffield, Sheffield, UK

**Keywords:** EQ-5D, generic health status measures, health-related quality of life, preference-based measures, quality of life, schizophrenia, SF-36, SF-12, SF-6D

## Abstract

**Objectives:**

Generic health status measures such as the short form health survey (SF-36) and EuroQol-5D (EQ-5D) are increasingly being used to inform health policy. They are claimed to be applicable across disease areas and have started to be used within mental health research. This review aims to assess the construct validity and responsiveness of four generic health status measures in schizophrenia, including the preference-based SF-6D and EQ-5D.

**Method:**

A systematic review of the literature was undertaken. Ten databases were searched from inception to August 2009 and reference lists scrutinized to identify relevant studies. Studies were appraised and data extracted. A narrative synthesis was performed of the evidence on construct validity including known groups validity (detecting a difference in health-related quality of life (HRQL) scores between two different groups such as samples from the general population and people with schizophrenia), convergent validity (strength of association between generic HRQL and other measures (e.g., symptom or functional), and responsiveness. Responsiveness was considered by: 1) differences in generic HRQL measure scores in responders/non-responders and 2) correlation between changes on generic HRQL measures and changes in specific measures obtained from patients and clinicians.

**Results:**

Thirty-three studies were identified that provided data on the validity and/or responsiveness of the instruments. Most of the evidence concerns the SF-36 and EQ-5D, and for these instruments there was evidence for known group validity. The evidence for convergent validity and responsiveness was mixed, with studies presenting contradictory results.

**Conclusion:**

Although the evidence base is limited in a number of important respects, including problems with the measures used to develop constructs in the validation studies, it is sufficient to raise doubts about the use of generic measures of health like the EQ-5D and SF-36 in patients with schizophrenia.

## Introduction

Generic health status measures such as short form health survey (SF-36) and EuroQol-5D (EQ-5D) are increasingly being used to inform health policy. The last decade has seen the increased use of economic evaluation, particularly the use of cost-effectiveness analyses by agencies such as National Institute for Health and Clinical Excellence (NICE) to inform resource allocation decisions [1], where interventions are assessed in terms of their cost per quality adjusted life year (QALY). The QALY provides a way of measuring the benefits of health care interventions, including improvements in health-related quality of life (HRQL) usually measured using a generic measure like EQ-5D. There has been, however, only a limited use of generic measures of health in mental health [2].

It is claimed that the EQ- 5D and other generic preference-based measures such as the SF-6D [3] are applicable to all interventions and patient groups. This claim has support in many physical conditions where these instruments have managed to pass psychometric tests of reliability and validity [4]. For other conditions the claim has not be substantiated, such as in relation to visual impairment in macular degeneration [5] and hearing loss [6]. Doubts have also been raised about the appropriateness of generic measures in mental health [7]. One solution would be to use disease-specific HRQL measures, for example there have been attempts to derive preference-based measures from the positive and negative syndrome scale (PANSS) and clinical outcomes in routine evaluation – outcome measure (CORE-OM) [8,9] in mental health. There are concerns, however, about the comparability of such disease-specific scales and in the United Kingdom, health technology assessment submissions to NICE are expected to follow the details outlined in the reference case analysis described by the NICE methods guide. This clearly stipulates that wherever possible and appropriate, the EQ-5D is the favored measure for generating utility values [1], thus allowing a common metric to assess health care interventions. Alternative measures may be used where the EQ-5D has been empirically demonstrated to be inappropriate in terms of their validity and responsiveness to change and several studies have been undertaken providing such evidence.

In order to provide a reasoned assessment of the appropriateness of generic HRQL measures in patients with schizophrenia, we have undertaken a systematic review to investigate the construct validity and responsiveness of two generic HRQL profile measures (SF-36, SF-12) and two preference-based HRQL measures (SF-6D, EQ-5D) in schizophrenia.

## Methods

### Measures being evaluated

The SF-36 is a generic health status profile measure consisting of eight dimensions of general health (GH); bodily pain (BP); physical functioning (PF); role-physical (RP), mental health (MH); vitality (V); social functioning (SF), and role-emotional (RE). These eight dimensions also can be used to generate a physical and mental health summary scores [10]. The SF-12 [11] is a shortened version of the SF-36, containing 12 of the SF-36 items, and also produces two weighted summary scores (PCS and MCS).

The EQ-5D valuation questionnaire comprises a five-dimensional questionnaire and an EQ-5D visual analogue scale (VAS). Respondents are asked to provide a position on the EQ-5D health state classification and to report their level of problems (no problems, some/moderate problems or severe/extreme problem) on the questionnaire, which includes mobility, self-care, usual activities, pain, and anxiety/depression. Responses can be converted into one of 243 different health state descriptions (ranging from no problems on any of the dimensions [11111] to severe problems on all five dimensions [33333]) and each one has its own preference-based score. Preference-based scores are determined by eliciting preferences: establishing which health states are preferred from a population sample. In order to do so, a method such as time trade off is used and involves asking participants to consider the relative amounts of time (for example, number of life-years) they would be willing to sacrifice to avoid a certain poorer health state [12]. Utility values from the UK EQ-5D can range from –0.59 to 1, where negative values are felt to be worse than death and a value of 1 indicates perfect health. The EQ-5D VAS reports on the respondent's self-rated valuation of his or her health stated; thus, it is based on the preferences of the patient, but is not preference based and not normally used to generate QALYs.

The SF-6D is a preference-based measure of health that can be generated from items of the SF-36 or SF-12 [3,13]. The SF-6D has a classification that describes health on six multilevel dimensions of physical functioning, role limitations, social functioning, pain, mental health, and vitality. There are algorithms for scoring each state based on values obtained from general population surveys using standard gamble (respondents make a series of choices which allow estimation of the strength of preferences regarding a health state). Health state utility values range from 0.29 to 1.0. These health state utility values can be used to calculate QALYs for cost-effectiveness analysis.

### Inclusion and exclusion criteria

Studies were eligible for inclusion if they contained HRQL data using one or more of the following instruments: SF-36, SF-12, SF-6D, or EQ-5D within the specified population: adults (≥18 years old) with schizophrenia or schizophrenia-related disorders (e.g., schizophreniform disorder or schizoaffective disorder). HRQL data could be from descriptive systems (i.e., their items and dimensions), health state utility values generated by the EQ-5D or SF-6D, or the EQ-5D VAS. Studies with the primary focus on individuals with alcohol and/or drug dependency with comorbid schizophrenia or schizophrenia-related disorder were excluded. The outcomes had to include data that allowed measurement of the construct validity (i.e., known groups or convergent) or the responsiveness of the HRQL instrument(s). Responsiveness data had to be in the form of effect sizes, standardized response means (SRMs), or correlation with change scores on symptom measures. Studies that only provided data on other psychometric properties such as reliability, face validity, and content validity were not included.

### Identification of studies

As part of a wider review of HRQL measures in mental health funded by the Medical Research Council (MRC), this review focused on the construct validity and responsiveness of the four generic HRQL measures within schizophrenia. Other reviews were carried out, each focusing on one mental health condition, as part of the wider review. A literature search was performed to identify relevant research for all mental health conditions being investigated within the wider review using database thesaurus and free text terms. Two sets of search terms were combined: terms for each of the four HRQL measures AND terms for the each mental health condition (search strategies are available from authors). Ten databases were searched for published research from inception: Cochrane Database of Systematic Reviews, Cochrane Central Register of Controlled Trials, NHS Economics and Evaluations Database, Health Technology Database, Database of Abstracts of Reviews of Effects, MEDLINE, PreMEDLINE, CINAHL, EMBASE, and Web of Science. Searches were limited to English language only, but not by date restriction. All searches were conducted in August 2009. The reference lists of relevant studies were searched for further articles.

Citations identified by the searching process were screened by one reviewer (DP) using the inclusion criteria. The full texts of articles were retrieved for any titles or abstracts that appeared to satisfy the inclusion criteria, or for which inclusion or exclusion could not be definitely determined. The same inclusion and exclusion criteria were used to assess full articles and any queries over inclusion were resolved by discussion and consensus between two reviewers (DP/JB).

### Data extraction

Data from all included trials were extracted using a form designed specifically for this review, and piloted on one paper [6]. Data extracted included: country of publication, type of disorder, study sample characteristics (numbers, age, gender), other measures used, mean scores on HRQL measures, type and method of validity assessment, type and method of responsiveness assessment, and validity and responsiveness data. Extractions were performed by one reviewer (DP). Where duplicate publications reported on similar data, the most complete and recent data were extracted.

### Quality assessment

There is no formal method for assessing the quality of these studies (i.e., there are no quality assessment checklists). The methods described by Fitzsimmons et al. [14] were used to evaluate HRQL data in their systematic review on the use and validation of HRQL instruments within older cancer patients. This included whether tests of statistical significance were applied, differences between treatment groups were reported (where applicable, e.g., in known groups validity), clinical significance discussed, and missing data were documented. We also report on response and completion rates where these are provided.

### Evidence synthesis and meta-analysis

Due to the large degree of heterogeneity between studies (including types of study designs, HRQL instruments, population characteristics and methods of determining construct validity, and responsiveness), it was not appropriate to perform meta-analysis. Analysis was by narrative synthesis and data were tabulated. All analyses were performed based on the HRQL instrument, with data analysis grouped by type of validity (convergent/discriminant or known groups) or responsiveness measured.

### Defining validity and responsiveness

#### Validity

Construct validity is defined as the extent to which an instrument measures the construct it is designed to measure and in the settings it is designed for [15,16]. Construct validity can be measured by known or extreme groups where in theory, in two groups who differ in a trait or behavior, one group is expected to score significantly higher or lower compared with the other group [16]. Care must be taken to ensure that the groups are hypothesized to have different scores and for preference-based measures care must be taken to ensure that patients and the general public would have clear preferences for one over the other [17]. Convergent validity assesses the relationship of the instrument of interest to other measures of the same construct to which it should be related [16]. Convergent validity is the correlation between two measures that in theory are associated. Again, the instrument being used to test convergence of EQ-5D and SF-6D must be a good indicator of the trait or behavior, such as another preference-based measure may be hypothesized to be likely to have a strong relationship to preferences. The strength of correlation between the two instruments was calculated using statistical tests (Pearson's product moment correlation or Spearman's rank correlation). We have used the following categories for evidence of correlation: >0.6, very strong; ≥0.5 to <0.6, strong; <0.5 to ≥ 0.3, moderate; and <0.3, weak. Statistical significance was also attached to correlations (*P* < 0.05).

#### Responsiveness

Walters [15] defined responsiveness as the extent to which an instrument can detect a clinically significant or practically important change over time. Any change must be perceptible and important to patients, and something that would be valued by the general public. Responsiveness can be measured in a number of ways by effect size statistics [15] standardized in different ways, such as dividing through by the SD at baseline or SD of the change in scores over time (i.e., standardized response means). Within this review, Cohen's [18] categories for magnitude of effect size were used: ≥ 0.80, large; <0.80 and ≥ 0.50, moderate; and 0.30 to <0.50, small.

The application of these psychometric criteria to preference-based measures requires some adaptation [17].The purpose of EQ-5D or SF-6D is to identify all differences or changes in health that are important to patients and valued by the general public. An item of the EQ-5D, for example, may fail to pick up small differences in one condition-specific dimension or miss another health dimension entirely, but if these are not important to patients and not valued by the general population, then it is not a weakness of the instrument. Equally, the EQ-5D may fail to reflect clinical differences, but these may not be important to patients. Thus, the tests of construct and convergent validity and responsiveness need to be applied with care.

## Results

### Study characteristics

**Fig. 1:**
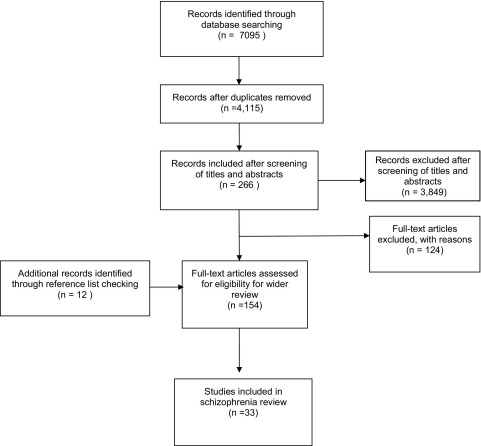
Flow diagram of study identification.

The search for studies for the wider review retrieved 4115 unique citations (Fig. 1). Of these, 3849 were excluded at the title and abstract stage and 266 full articles were examined. Another 12 studies were identified through reference list checking. Thirty-three studies were identified that provided data on the validity and/or responsiveness of the EQ-5D, SF-36, SF-12, or SF-6D (Tables 1–3 and Appendix found at doi:10.1016/j.jval.2011.04.006) within individuals diagnosed with schizophrenia, schizophreniform disorder, or schizoaffective disorder. Six studies were undertaken internationally across more than one country [8,19–23]; six studies were undertaken in the USA [24–29]; three in Germany [30–32]; two in Ethiopia [33,34]; two in France [35,36]; two in Spain [37,38]; two in the Netherlands [39,40]; and two in the United Kingdom [41,42]. The remaining six studies took place in Australia [43], Canada [44], Denmark [45], Hong Kong [46], Italy [47], and Poland [48]. Two studies did not report the country in which the study was undertaken [49,50].

The number of participants in the studies with schizophrenia or related conditions ranged from 15 to 2657. Participants included both genders and the proportions are reported in Tables 1, 2, and Appendix found at doi:10.1016/j.jval.2011.04.006. The mean age of participants with a schizophrenia spectrum disorder, reported in 24 of the 33 studies, ranged between 20.3 and 57.9 years. Three studies provided an age range of participants, but not a mean age [19,29,39]. Six studies did not provide information on age [21,33,37,43,44,49].

All studies obtained HRQL information from patients: seven studies compared patient HRQL values with published general population “normative” values [22,23,25,28,33,34,45]; three compared HRQL values with normal comparison participants that were recruited to the study [24,29,32]; and two used “norms” from healthy participants who had taken part in large surveys [43,46].

### Quality of included studies

Quality assessment of the studies was restricted to items relating to the quality of HRQL reporting and methods used in HRQL data analysis as previously described (Table 4). All but four studies reported tests for statistical significance of the properties measured [33,43,45,49]. Twelve of the 20 studies where it was applicable reported that tests were undertaken for difference between groups (e.g., known groups validity, responsiveness). Nine of the 33 studies considered what constituted a clinically significant difference in HRQL scores (Table 4), either by providing a predefined value or discussing whether the results were clinically meaningful. However, there was no discussion or inclusion of clinical significance defined in terms of patient perception; thus, from the perspective of preference-based measures, the lack of patient preference undermines the concept of clinical significance. Only three studies fully reported missing HRQL data and four studies partly reported this information. This has implications for the representativeness of these samples due to possible selection bias.

### EQ-5D

Seven studies examined the convergent validity of the EQ-5D [20,30,31,35,38,41,47] and one study examined the construct validity of the EQ-5D by the known groups method [41] (Table 1). Four studies investigated the responsiveness of the EQ-5D [20,40,41,49]. Seven studies investigating the EQ-5D used population preferences to generate an index value [20,30,31,38,40,41,49].

#### Known groups validity

Barton et al. [41] demonstrated known groups validity for the EQ-5D index whose scores differed according to the severity of disease. Clinically significant differences in EQ-5D index scores (defined as >0.03) were found between individuals defined as “severe” or “less severe” on seven symptoms or functioning measures, which included the PANSS, Hamilton depression rating scale, and global assessment of functioning (GAF).

#### Convergent validity

##### Symptom measures

Correlation with the EQ-5D and measures of symptoms or symptom severity such as the PANSS, symptom checklist-90-revised (SCL-90R), clinical global impression severity of illness scale (CGI-S), and brief psychiatry rating scale (BPRS) were modest or occasionally strong in three studies [20,30,38]. Two studies, however, found associations with the PANSS measures as nonexistent or mostly weak [41,47]. Moderate to strong associations between EQ-5D index scores and depression or anxiety symptom measures were recorded in one study [41].

##### Functioning and other quality of life measures

Association with the functioning measure, GAF, was mixed – it was non-existent in one study [41] and moderate or strong in two studies [30,38]. Similar association between the EQ-5D index and the social and occupational functioning assessment scale (SOFAS) was non-existent in one study [41], and it was moderate with UK and German versions of the EQ-5D index (0.44 and 0.42, respectively, *P* < 0.001) [30]. The EQ-5D index was moderately to strongly associated with the health of the nation outcome scales (HoNOS) and weakly to moderately correlated with the global assessment of relational functioning (GARF); whereas EQ-5D health state scores were mostly moderately to strongly associated with these measures) [30].

Most moderate and significant correlations were found between the EQ-5D descriptive system health states score and the schizophrenia quality of life questionnaire (S-QoL) [35]. Barton et al. [41] found no association between the EQ-5D index and another schizophrenia-specific HRQL measure, the quality-of-life scale (QLS) [41]. Konig et al. [30] found no association between the EQ-5D index and direct utilities elicited by the time trade off method.

#### Responsiveness

Responsiveness data were also mixed. Two studies demonstrated that the EQ-5D VAS and EQ-5D index were responsive to change in patients [41,49]. There was weak evidence to suggest that EQ-5D index scores were associated with changes >25% on the BPRS and there was little or no association found when changes on the BPRS were <25% [20]. Furthermore, van de Willige et al. [40] found that EQ-5D index scores did not respond to changes in most symptom or functioning measures, only showing significant (but not always moderate or strong) correlations with the PANSS positive subscale, auditory hallucinations rating scale (AHRS), and Groningen social disabilities schedule (GSDS) (–0.39, *P* < 0.005). The EQ-5D dimension and VAS scores appeared to perform better than the EQ-5D index in the same study [40].

### SF-36

Fourteen studies examined the construct validity of the SF-36 using convergent validity [8,21,23,28,32,35–37,39,42,44,46–48] and 12 studies examined the construct validity of the SF-36 using known groups validity [19,22–25,28,29,32–34,45,46]. Nine studies investigated the responsiveness of the SF-36 [21,26,27,32,34,36,44,48,50]. (See Table 2 for concise version of SF-36 validity and responsiveness evidence and Appendix found at doi:10.1016/j.jval.2011.04.006 for further details on the evidence for the validity and responsiveness of the SF-36.).

#### Known groups validity

Eleven studies compared SF-36 scores with normative values. Normative values were taken mostly from general population samples and published figures, although some studies recruited a sample of “normal participants” to compare SF-36 scores [24,29,32]. Almost all studies found statistically significant differences in SF-36 summary (PCS and MCS) and dimension scores between individuals with schizophrenia and normative values; this could be up to 80 points in difference on the MCS and its dimensions and up to 50 points in difference on the PCS and its dimensions. Two exceptions were Sciolla et al. [29] and Norholm et al. [45], where statistically significant differences were noted for all dimensions except bodily pain.

One study investigated the effect of the presence of side effects on SF-36 scores. Scores were between two and five points lower on the PCS and MCS for individuals with some side effects (e.g., subjective rigidity or anticholinergic effect) when compared with those who did not have those side effects; these differences were statistically significant [19]. This was not the case, however, for all side effects; for example the MCS and PCS did not differ between participants presenting with subjective akathisia and weight gain (among others).

#### Convergent validity

##### Symptom measures

Five studies found mostly weak or non-existent correlations with symptom measures such as the PANSS, Scale for the Assessment of Negative Symptoms (SANS), Extrapyramidal Symptom Rating Scale (ESRS), BPRS, and CGI-S [23,32,36,46,47]. There was some evidence of stronger association with the PANSS in two studies [44,48] and the BPRS in another study [28]. Correlations with measures of depression such as the Montgomery-Åsberg depression rating Scale (MADRS) and Calgary depression *s*cale for schizophrenia (CDSS) were weak in two studies [28,46] and moderate to strong in another study [23].

##### Functioning and other quality-of-life measures

Correlation with the GAF was recorded as weak to moderate in one study [39] and very strong in another study [44]. The SOFAS was correlated very strongly with the SF-36 [44]. Strong and statistically significant (*P* < 0.001) correlations were reported with two schizophrenia-specific HRQL measures [35,42]. Revicki et al. [21] described very weak correlations between the SF-36 and schizophrenia-specific QLS total score. Correlations with generic HRQL measures like the World Health Organization quality of life instruments (WHOQoL-BREF, WHOQoL-100, and WHOQoL-26) were mostly moderate to very strong (i.e., >0.3) [37,46].

#### Responsiveness

Little evidence existed to demonstrate that when changes were recorded on the PANSS, this correlated with changes on the SF-36, with the association being mostly weak and nonsignificant in four studies [26,27,48,50]. Pyne et al. [27] also found weak correlations with changes on the CDSS (–0.27, *P* < 0.01) and the extrapyramidal symptoms rating scale (ESRS) (–0.22, *P* < 0.05).

Responsiveness was also measured with other measures or by methods other than calculating correlation between change scores, but similarly this evidence was weak. Effect sizes calculated for patients judged to have improved or not improved according to CGI-S scores were all nonsignificant apart from for social functioning, which was small in size [36]. Milliken et al. [44] found higher MCS scores in remitted versus nonremitted participants, but this was only a trend and not statistically significant (*P* = 0.063). Revicki et al. [21] reported that the total MCS indicated statistically significant contributions for changes in the PANSS positive scale and the MADRS. Although Pukrop et al. [32] found that improvement in negative symptoms significantly impacted the role physical and role emotional dimensions (and also remained significant when controlling for improvement in negative symptoms), no such interactions remained significant for any dimensions when controlling for improvement in positive symptoms. However, Kebede et al. [34] found that SANS and Scale for the Assessment of Positive Symptoms (SAPS) scores were inversely related with improvements in physical and social functioning domains and role limitations due to emotional problems.

### SF-12

Data were limited to one study containing known groups validity evidence, and revealed that individuals with psychosis were significantly (*P* < 0.001) more likely to report disability on the SF-12 than individuals with no mental health disorder [43] (Table 3).

### SF-6D

Data were limited to one study that demonstrated moderate correlation between the SF-6D index and the symptom measure BPRS (–0.344, no *P* value) [20] (Table 3). When changes occurred on the BPRS, however, changes in the SF-6D were correlated only weakly (–0.22, no *P* value) and appeared only able to respond to changes on the BPRS greater than 25%. Data for the SF-6D scores were normally distributed, thus there was no evidence for floor or ceiling effects.

### Distributional properties of the measures

Only five studies reported distributional properties of the measures: three for the EQ-5D [20,30,38]; and one study each for the SF-36 [23] and SF-6D [20]. Scores were found to be normally distributed for the SF-36 [23] and SF-6D [20]; thus, there was no evidence of floor or ceiling effects. The three studies which report on the distributional properties of the EQ-5D [20,30,38], however, found that the EQ-5D index showed a moderate ceiling effect (for example, Konig et al. reported 21% of respondents achieved the maximum score) [30]. This ceiling effect could potentially limit the responsiveness of the measure. In contrast, two of the three studies found that the EQ-5D VAS was normally distributed [30,38].

## Discussion

Thirty-three studies were identified that examined the validity and/or responsiveness of four generic HRQL measures, although very limited data were found for the generic health status measure SF-12 and the preference-based SF-6D. The studies were undertaken in a variety of countries, mostly in Europe and North America, illustrating the wide use of such measures internationally.

The majority of the evidence (25 studies) examined the validity and responsiveness of the SF-36. Although there appears to be strong evidence that the SF-36 is able to distinguish between general population norms and scores of people with schizophrenia (known groups validity), the evidence for convergent validity and responsiveness is less certain. Similar findings existed for the EQ-5D, with mixed evidence for the properties of convergent validity and responsiveness. Indeed, when strong associations were found between individual EQ-5D health state dimensions (e.g., anxiety/depression or self-care) and symptom or functioning measures, this did not necessarily translate into comparable changes in overall EQ-5D index scores such as utility values [30,40]. For psychiatric research, it may be that the physical health domains are overly stressed and with less emphasis on mental health, the total EQ-5D index scores may not be accurately represented [40].

There was some evidence that associations with measures of depression were comparatively stronger than those with symptom measures of schizophrenia (e.g., PANSS) [23,30,36,41]. This may indicate that the generic HRQL measures were only able to detect this component of HRQL or that depression is the only component of HRQL within schizophrenia that is important within the context of HRQL measurement. The issue is whether schizophrenia has quality-of-life implications not adequately described by the five dimensions of the EQ-5D.This is an important issue that needs to be explored further using a range of research methods, including qualitative interviews with patients.

### Types of measures

When testing association between measures for convergent validity (or change scores in responsiveness), there are good reasons to predict that stronger and more consistent correlations might exist between generic HRQL measures and functioning (e.g., GAF, SOFAS) or mental health/schizophrenia-specific HRQL (e.g., QLS) measures than purely symptom-based measures such as the PANSS. These types of measures are more likely to measure similar concepts to that of generic HRQL measures and due to this degree of overlap, we could reasonably assume that these measures would correlate well with generic HRQL measures. By their very nature, symptom measures are measuring different concepts to HRQL measures, so it might be reasonable to predict that it is less likely that a strong correlation might exist. Similarly, one might expect a greater degree of association between subjective measures (completed by patients) and generic HRQL measures than with objective symptom measures (typically completed by clinicians).

Re-examining the evidence accounting for the type of measure used to assess convergent validity (symptom vs. functioning or HRQL measures; subjective vs. objective measures), for whichever the type of measure the evidence for convergent validity remains uncertain in this population. Ten studies suggested no or uncertain evidence for a correlation between symptom measures and generic HRQL measures [8,20,23,32,36,41,46–48] whereas four revealed moderate to strong correlations [28,30,41,44]. Functioning and schizophrenia HRQL measures did not fare much better, with four studies indicating strong evidence for convergent validity [35,38,42,44] and four describing uncertain or no evidence of such a relationship [21,36,39,41]. Of the seven studies that used objective measures to test an association, four reported a strong evidence for convergent validity [35,37,42,46] and three found no such evidence [30,31,41].

Thus, it seems there is a wider issue regarding what types of measures might reasonably be expected to correlate strongly with generic HRQL measures. It is difficult to determine how strongly correlated in theory generic HRQL measures should be with symptom and/or other measures and there is little guidance on what constitutes reasonable correlation. Indeed, Walters [15] noted that some would say it is impossible to *prove* validity of HRQL instruments because no “gold standard” exists. Although a number of different concepts or constructs will be the same or similar between HRQL and other measures, there will of course be some areas where there is no overlap. Also, as discussed previously, where health dimensions and changes appear to have been missed by preference-based HRQL measures, these may not actually be important to patients or valued by the general population; thus it cannot be determined as a weakness of the measure. This needs to be explored in further research.

### Strengths and limitations

This review comprehensively identified studies that reported on the construct validity and responsiveness of four generic HRQL measures (SF-6D, SF-12, SF-36, and EQ-5D), and then tabulated and provided a narrative synthesis of the findings. The review has some limitations, mainly due to compromising on some elements of the review process due to the large scope of the project. The search for studies was reasonably comprehensive, but it was limited to key databases and reference list checking of included studies, and study selection was undertaken by one reviewer. Ideally, further searching could be undertaken in trial registries, conference proceedings, and by citation searching to make the search process fully comprehensive. Study quality assessment has been pragmatic and focused on the elements that contribute to HRQL analysis. The populations included in this review were heterogeneous in terms of the nature of schizophrenia (e.g., clinical form, evolution form, medication), but not all studies provided detailed or uniform information on these characteristics. Such clinical variables clearly have an impact on HRQL and these factors will have had an impact upon the results of individual studies. Nevertheless, this review gives an overall picture of the validity and responsiveness of these four measures in this population and provides a starting point for future more focused reviews and future primary research.

### Further research

There is very limited evidence of validity or responsiveness for the SF-12 and SF-6D and, though they are derivatives of the SF-36, they have a limited item coverage (12 and 11, respectively) and may not perform as well. Therefore, further research needs to be directed toward demonstrating these properties for these instruments.

Research also needs to be directed toward developing robust methods of demonstrating validity and responsiveness for generic HRQL measures. For known groups validity, the evidence discriminating between healthy and not healthy individuals could be considered fairly crude; large differences should be obviously apparent between such groups. Therefore, research is required to demonstrate an instrument that can reflect these differences between different severities of the disorder. For convergent validity, this might mean consideration of which measures to choose for assessment of strength of correlation, both by considering the type of measure (e.g., symptom functioning or HRQL) and the nature of measure (subjective or objective). Studies need to be explicit at their outset about the hypothesized associations when investigating validity and responsiveness. In addition, wherever studies can investigate feasibility of generic HRQL measures alongside construct validity and responsiveness within this disease area, this will allow a greater overall understanding of which measures are useful within schizophrenia.

This review was limited to examining quantitative evidence, but researchers and regulatory authorities, such as the Food and Drug Administration and the European Medicines Agency, also require qualitative evidence on the validity of measures in specific patient groups based on interviews with patients [51,52]. Some of the questions raised in this review might be better addressed through the use of qualitative interviews with patients who will provide greater insight into the shortcomings of these generic measures. Whereas qualitative research indicates generic measures, such as the EQ-5D and SF-6D, are not suitable within schizophrenia, alternative HRQL measures need to be found. One alternative is to develop a preference-based mental health index based on either an existing measure or the development of a new one [53]. Another alternative would be to develop “add-on” dimensions that reflect the concerns of patients with mental health conditions like schizophrenia.

## Conclusion

In conclusion, the evidence found in this review on the validity and responsiveness for a number of widely used generic measures in patients with schizophrenia has been mixed. Although the evidence base is limited in a number of important respects (including problems with the measures used to develop constructs in the validation studies), it is sufficient to raise doubts about the use of generic measures of health like the EQ-5D and SF-36 in patients with schizophrenia. This suggests that agencies, such as NICE, which advise on reimbursement of health costs, should be willing to consider evidence on health state utility values based on other methods.

## Figures and Tables

**Table 1:** EQ-5D validity and responsiveness.

Study	Population characteristics	Properties measured	Source and types of measures used to test convergent validity and/or responsiveness⁎	Details of validity or responsiveness
Auquier (2003) [35] France	DSM-IV schizophrenia Inpatients and outpatients (numbers not reported). N=207 (141 males and 66 females).Mean age, 37.3 (SD, 10.9) (range 18–70 years).	Convergent validity	Patient–completed i) Quality of life-disease specific (S-QOL)	Correlations with EQ-5D descriptive system health states and SQoL dimensions ranged from 0.06 (SQoL family relationships) to 0.56 (SQoL self-esteem). Generally moderate correlations, overall correlation with S-QOL index was moderate and significant: 0.48, *P* < 0.05
Badia (1999) [49] Country not reported	Schizophrenia (classification not reported). N=approx 2949 (n=2128 olanzapine; n=821 risperiodone or haloperidol; small numbers on other antipsychotics). No age, gender or inpatient/outpatient status reported.	Responsiveness	No measures reported	EQ-VAS and EQ-5D index recorded large effect sizes (0.98 and 1.13, respectively) for olanzapine-treated patients pre- and post-treatment and moderate to large effect sizes for other antipsychotics (0.58 to 0.75 for VAS and 0.78 to 0.96 for index).
Barton (2009) [41] UK	Non-affective psychosis diagnosis (criteria not specified). Includes: schizophrenia, schizoaffective disorder, bipolar disorder, and psychotic depression. Participants had to screen positive for psychotic symptoms and in relative remission (≤4 on PANSS). N=77 (55 males, 22 females). Mean age, 28.9 years; range 18–52.50/77 had a diagnosis of non-affective psychosis. Inpatient/outpatient status not reported.	Known groups validity. Convergent validity. Responsiveness	Clinician-completed i)Symptoms PANSS ii)Functioning GAF, SOFAS iii)Quality of life-disease specific QLS (interviewer administered) Patient-completed i) Symptoms BAI, BDI, BHS	EQ-5D Index scores showed at least a minimally important clinical difference (MID) (defined as >0.03) between those with milder and more severe scores on symptom and functioning measures. Correlations between the EQ-5D index and three symptom measures (BAI, BDI, BHS) were moderate to very strong (0.360–0.656). A significant but weak correlation was found with a measure the GAF(0.263). Non-significant and weak correlations were seen with the PANSS, QLS and SOFAS. Mean EQ-5D scores were higher for those who improved than those who did on improve on 6 of 7 symptom or functioning measures. The difference in means between improvers and non-improvers was equal to or greater than the MID (0.03).
Konig (2007) [30] Germany	ICD-10: Schizophrenia, schizotypal or delusional disorders. 49.4% outpatient; 41.6 % inpatient; 9.0% day clinic. N=166 (97 males, 69 females). Mean age, 40.5 (SD, 11.1); range 21–80 years.	Convergent validity	Clinician-completed i) Symptoms PANSS, SCL-90R & CGI-S ii) Functional GAF, GARF, SOFAS & HoNOS Patient-completed i) Quality of life-generic TTO direct utility & WHOQOL-BREF	Effect sizes (calculated using the mean values of symptom and functioning measures between individuals who answered “yes” or “no” for each EQ-5D dimensions) were mostly moderate to large for symptom measures (0.37–1.29) and functioning measures (0.24–1.4). Effect sizes for the for the pain/discomfort dimension were smaller. Moderate correlations recorded between EQ-5D VAS and index and symptom measures (0.34–0.73), functioning measures (0.20–0.65), and generic quality of life measures (0.47–0.57).
Konig (2009) [31] Germany	ICD-10: Schizophrenia, schizotypal or delusional disorders.51.7% outpatient; 38.5% inpatient, and 9.8 day clinic. N=143 (83 males and 60 females). Mean age, 40.4 (SD, 11.6).	Convergent validity	Clinician-completed i) Symptoms PANSS, SCL-90R, CGI-S, and BRAMES ii) Functional GAF, GARF, SOFAS, and HoNOS Patient-completed ii) Quality of life-generic TTO direct utility & WHO-QOL-BREF	Correlation with the TTO direct elicitation of utility values and the EQ-5D VAS and EQ-5D index (UK and German) were weak in correlation (0.25). However, the TTO method did not correlate well with a number of theoretically related measures.
McCrone (2009) [20] The Netherlands, Germany, UK, and Italy	SCAN interview diagnosed schizophrenia (classification scheme not specified).“Chronic high disability sample” based on number of years on medication, number of psychiatric inpatient days last year, and GAF score. N=409 (245 males and 164 females). Mean age, 41.5 (SD, 11.5); no range reported.	Convergent validity. Responsiveness	Clinician-completed i) Symptoms BPRS	Moderate correlation (0.343) with EQ-5D index and a symptom measure (BPRS) at baseline. Weak correlation (0.29) with changes in symptom measure after treatment. Where improvement on BPRS was at least 25%, EQ-5D SRM was small in size (0.39). Where deterioration on BPRS was at least 25% or improvement on BPRS <25%, EQ-5DSRMs were very small (0.17 and 0.05 respectively).
Prieto (2004) [38] Spain	ICD-10 Schizophrenia. N=2657 (1691 males and 966 females). Not stated if inpatient or outpatient N=2128 on olanzapine; n=417 on risperidone; n=112 on haloperidol. Mean age, 35.32 (SD, 11.57); range not reported.	Convergent validity	Clinician-completed i) Symptom CGI-S ii) FunctionalGAF	EQ-5D index and EQ-5D VAS both demonstrated moderate to strong association with one symptom (CGI-S) and one functional measure (GAF), range 0.34–0.54, *P* < 0.001).
Scalone (2008) [47] Italy	N= 637 (n=551with schizophrenia n=86 with schizophreniform disorder). 414 males and 223 females; 18–40 years old (no mean age reported). Inpatient/outpatient status not reported.	Convergent validity	Clinician-completed i) Symptom PANSS, CGI-S ii) Functional GAF	Weak to moderate correlations between QOL scores (EQ-5D and SF-36) and symptom measures (PANSS and CGI-S) ranging from 0.189–0.393.
van de Willige (2005) [40] The Netherlands	DSM-IV schizophrenia (described as chronic sample). Auditory hallucinations for > 2 years after adequate treatment. Use of at least 2 antipsychotic drugs. Inpatients and outpatients-numbers not reported. N=76 (42 males and 34 females). Mean age, 36 years (SD, 11.2).	Responsiveness	Clinician-completed i) Symptom PANSS, AHRS ii) Functional GSDS iii) Quality of life-generic WHOQOL-BREF	Differences in EQ-5D descriptive system scores between baseline and follow-up were statistically significant for the daily functioning domain (Z=1.79, *P* > 0.05 < 0.10) and anxiety/depression domain (Z=3.53, *P* < 0.001). Moderate correlations between changes on EQ-5D VAS and changes in PANSS (total and subscales) (0.34–0.47, *P* < 0.01 and *P* < 0.0005). Correlations between changes on EQ-5D index and changes in PANSS existed only on PANSS positive symptoms subscale (0.53, *P* < 0.001). Moderate to strong correlations with 3 of 4 AHRS subscales and the EQ-5D VA (0.46–0.50, *P* < 0.001). The EQ-5D index was only correlated with one AHRS subscale and this was weak (distress, 0.25, *P* < 0.01). Moderate correlations with social function (GSDS) on both the EQ-5D VAS (0.27–0.46, *P* ranges < 0.01 and < 0.001) and EQ-5D index (0.29–0.39, *P* ranges < 0.05 and < 0.005). WHOQoL-Bref dimensions correlated for the most part moderately to strongly with the EQ-5D VAS (0.27–0.60) and EQ-5D index (0.25–0.58).

AHRS, auditory hallucinations rating scale; BAI, Beck anxiety inventory; BDI, Beck depression inventory; BHS, Beck hopelessness scale; BPRS, brief psychiatry rating scale; BRAMES, Bech–Rafaelsen melancholia scale; CDSS= Calgary depression scale for schizophrenia; CGI-S, clinical global impression-severity; EQ-5D, EuroQol-5D; ESRS, extrapyramidal symptom rating scale; GAF, global assessment of functioning; GARF, global assessment of relational functioning scale; GSDS= Groningen social disabilities schedule; HoNOS, health of the nation outcome scales; PANSS, positive and negative syndrome scale; QLS = quality of life scale; QoLI, quality of life inventory; SCL-90R, symptom checklist-90-R; SF-36, short form health survey; SOFAS, social and occupational functioning assessment scale; S-QOL, schizophrenia quality of life questionnaire; TTO, time trade off; VAS, visual analogue scale; WHO-QOL-BREF= WHO quality of life-BREF.

⁎ Note: other measures used in the study, but not used to test convergent validity or responsiveness, are not listed.

**Table 2:** Summary of evidence for SF-36 by property (more detailed evidence is presented in the Appendix found at doi:10.1016/j.jval.2011.04.006)

Number of studies	√	?	X
Known groups validity	11	1	0
Convergent validity	7	2	5
Responsiveness	1	2	5

KEY

√ Evidence suggests property exists (e.g., statistically significant difference in scores for known groups validity or moderate to strong correlations for convergent validity).

? Mixed evidence for property.

X Evidence suggests property does not exist (e.g., weak correlations for convergent validity).

**Table 3:** SF-12 and SF-6D validity and responsiveness.

Study	Patient characteristics	Properties measured	Source and types of measures used to test convergent validity and/or responsiveness*	Details of validity or responsiveness
SF-12 validity and responsiveness				
Sanderson (2002) [43] Australia	DSM-IV psychosis (not defined). 50 participants (male/female not provided). No mean age or range reported. Inpatient/outpatient status not reported.	Known groups validity	Not applicable	Linear regression demonstrated that individuals with psychosis were significantly (*P* < 0.001) more likely to report disability on the SF-12 than individuals with no mental health disorder. SF-12 scores were around 12 points lower in individuals with psychosis.
SF-6D validity and responsiveness				
McCrone (2009) [20] The Netherlands, Germany, UK, and Italy	SCAN interview diagnosed schizophrenia (classification scheme not specified). “Chronic high disability sample” based on number of years on medication, number of psychiatric inpatient days last year, and GAF score. N=409 (245 males and 164 females). Mean age, 41.5 (SD, 11.5); no range reported.	Convergent validity. Responsiveness	Clinician-completed ii) Symptoms BPRS	Moderate correlation (0.314) with a symptom measure (BPRS) at baseline. Weak correlation (0.22) with changes in symptom measure after treatment. Where improvement on BPRS was at least 25%, SRM was moderate in size (0.39). Where deterioration on BPRS was at least 25% or improvement on BPRS <25%, SRM was very small (0.27 and 0.02, respectively).

BPRS, brief psychiatry rating scale; GAF, global assessment of functioning; SF-6D, short form 6D (preference-based) generated from items of the SF-36 or SF-12; SF-12, short form 12 (shortened SF-36); SRM, standardized response mean.

**Table 4:** Quality assessment of included studies.

Study details	Properties measured	Statistical significance tested for properties measured	Difference between groups	Clinical significance addressed or discussed	Missing HRQL data documented ⁎
Auquier (2003) [35]	Convergent validity	Yes	Not applicable	Not reported	Not reported (for SF-36)
Badia (1999) [49]	Responsiveness	Not reported	Not reported	Not reported	Not reported
Barton, G (2009) [41]	Known groups and convergent validity. Responsiveness	Yes	Not reported	Yes	Partly – numbers presented for each analysis which demonstrate some non-completion, but no detail on EQ-5D completion.
Bebbington (2009) [19]	Known groups validity	Yes	Not reported but demographics adjusted for in analysis	Not reported	Partly – SF-36 domains were scored if participants completed 50% of a domain. Numbers varied between dimensions. However, we are not told how complete each dimension is.
Bobes (1997) [37]	Convergent validity	Yes	Not applicable	Not reported	Not reported
Dunayevich (2007) [50]	Responsiveness	Yes	Yes	Yes	Not reported
Folsom (2009) [24]	Known groups validity	Yes	Yes	Not reported	Not reported
Jarema (2001) [48]	Convergent validity Responsiveness	Yes	Not applicable	Not reported	Not reported
Kebede (2004) [33]	Known groups validity	Not reported	Not reported	Not reported	Not reported
Kebede (2005) [34]	Known groups and convergent validity.	Yes	Yes	Not reported	Not reported
Konig (2007) [30]	Convergent validity	Yes	Not applicable	Not reported but floor and ceiling effects are discussed.	Yes
Konig (2009) [31]	Convergent validity	Yes	Not applicable	Not reported	Partly – states some missing values for some variables and such patients are excluded. Does not state what EQ-5D values are missing.
Law (2005) [46]	Known groups and convergent validity	Yes	Yes	Not reported	Not reported
Lenert (2005) [8]	Convergent validity	Yes	Not applicable	Not reported	Not reported
McCrone (2009) [20]	Convergent validity and responsiveness	Yes	Not applicable	Yes	Yes
Meijer (2002) [39]	Convergent validity	Yes	Not applicable	Not reported	Yes
Milliken (2007) [44]	Convergent validity and responsiveness	Yes	Not reported	Not reported	Not reported
Nasrallah (2004) [25]	Known groups validity and responsiveness	Yes	Yes	Yes	Not reported
Norholm (2007) [45]	Known groups validity	Not reported but age-matched sample used to compare scores	Yes	Not reported	Not reported
Phillips (2006) [26]	Convergent validity and responsiveness	Yes	Yes	Not reported	Not reported
Prieto (2004) [38]	Convergent validity	Yes	Not reported	Not reported but ceiling effects discussed	Not reported
Pukrop (2003) [32]	Known groups and convergent validity and responsiveness	Yes	Yes	Not reported	Not reported
Pyne (2003) [27]	Responsiveness	Yes	Not applicable	Yes	Not reported
Reine (2005) [36]	Convergent validity and responsiveness	Yes	Not applicable	Yes	Not reported
Revicki (1999) [21]	Convergent validity and responsiveness	Yes	Yes	Yes	Not reported
Russo (1998) [28]	Known groups and convergent validity	Yes	Not reported	Not reported	Not reported
Sanderson (2002) [43]	Known groups validity	Not reported	Yes	Not reported	Not reported
Scalone (2008) [47]	Convergent validity	Yes	Not applicable	Not reported	Not reported
Sciolla (2003) [29]	Known groups validity	Yes	Yes	Not reported	Not reported
Strakowski (2005) [22]	Known groups validity	Yes	Not reported	Not reported	Not reported
Tunis (1999) [23]	Known groups and convergent validity and responsiveness	Yes	Yes	Yes	Partly – missing SF-36 values were mentioned by authors but actual percentages were not reported.
van de Willige (2005) [40]	Responsiveness	Yes	Not applicable	Yes	Not reported
Wilkinson (2000) [42]	Convergent validity	Yes	Not applicable	Not reported	Not reported

⁎ Actual missing values from instrument NOT lost to follow-up.

## References

[1] National Institute for HealthClinical Excellence (NICE) Guide to the methods of technology appraisal. http://www.nice.org.uk/aboutnice/howwework/devnicetech/technologyappraisalprocessguides/guidetothemethodsoftechnologyappraisal.jsp.

[2] Gilbody S., House A., Sheldon T. (2003). Outcome measures and needs assessment tools for schizophrenia and related disorders. Cochrane Database of Syst Rev.

[3] Brazier J., Roberts J., Deverill M. (2002). The estimation of a preference based measure of health from the SF-36. J Health Econ.

[4] Marra C.A., Ahidi A.A., Uh D. (2005). Are indirect utility measures reliable and responsive in rheumatoid arthritis patients?. Qual Life Res.

[5] Espallargues M., Czoski-Murray C., Bansback N. (2005). The impact of age related macular degeneration on health state utility values. Invest Ophthalmol Vis Sci.

[6] Barton G.R., Bankart J., Davis A.C., Summerfield Q.A. (2004). Comparing utility scores before and after hearing-aid provision. Appl Health Econ Health Policy.

[7] Brazier J. (2010). Is the EQ–5D fit for purpose in mental health?. Br J Psychiatry.

[8] Lenert L.A., Rupnow M.F., Elnitsky C. (2005). Application of a disease-specific mapping function to estimate utility gains with effective treatment of schizophrenia. Health Qual Life Outcomes.

[9] Mavranezouli I., Brazier J., Young T.A., Barkham M. (2010). Using Rasch analysis to form plausible health states amenable to valuation: the development of the CORE-6D from a measure of common mental health problems (CORE-OM). Qual Life Res.

[10] Ware J.E., Kosinski M., Keller S.D. (1994). SF-36 Physical and Mental Health Summary Scales: a User's Manual.

[11] Ware J.E., Kosinski M., Keller S.D. (1996). A 12-item short-form health survey: construction of scales and preliminary tests of reliability and validity. Med Care.

[12] Tolley K. What are health utilities?. http://www.medicine.ox.ac.uk/bandolier/painres/download/whatis/Health-util.pdf.

[13] Brazier J., Roberts J. (2004). Estimating a preference-based index from the SF-12. Med Care.

[14] Fitzsimmons D., Gilbert J., Howse F. (2009). A systematic review of the use and validation of health-related quality of life instruments in older cancer patients. Eur J Cancer Care.

[15] Walters S.J. (2009). Quality of Life Outcomes in Clinical Trials and Health-Care Evaluation: a Practical Guide to Analysis and Interpretation.

[16] Streiner D.L., Nomran G.R. (2003). Health Measurement Scales: a Practical Guide to Their Development and Use.

[17] Brazier J., Deverill M. (1999). A checklist for judging preference-based measures of health related quality of life:learning from psychometrics. Health Econ.

[18] Cohen J. (1988). Statistical Power Analysis for the Behavioral Sciences.

[19] Bebbington P.E., Angermeyer M., Azorin J.M. (2009). Side-effects of antipsychotic medication and health-related quality of life in schizophrenia. Acta Psychiatrica Scandinavica.

[20] McCrone P., Patel A., Knapp M. (2009). A comparison of SF-6D and EQ-5D utility scores in a study of patients with schizophrenia. J Ment Health Policy Econ.

[21] Revicki D.A., Genduso L.A., Hamilton S.H. (1999). Olanzapine versus haloperidol in the treatment of schizophrenia and other psychotic disorders: quality of life and clinical outcomes of a randomized clinical trial. Qual Life Res.

[22] Strakowski S.M., Johnson J.L., Delbello M.P. (2005). Quality of life during treatment with haloperidol or olanzapine in the year following a first psychotic episode. Schizophr Res.

[23] Tunis S.L., Croghan T.W., Heilman D.K. (1999). Reliability, validity, and application of the medical outcomes study 36-item short-form health survey (SF-36) in schizophrenic patients treated with olanzapine versus haloperidol. Med Care.

[24] Folsom D.P., Depp C., Palmer B.W. (2009). Physical and mental health-related quality of life among older people with schizophrenia. Schizophr Res.

[25] Nasrallah H.A., Duchesne I., Mehnert A. (2004). Health-related quality of life in patients with schizophrenia during treatment with long-acting, injectable risperidone. J Clin Psych.

[26] Phillips G.A., Van Brunt D.L., Roychowdhury S.M. (2006). The relationship between quality of life and clinical efficacy from a randomized trial comparing olanzapine and ziprasidone. J Clin Psych.

[27] Pyne J.M., Sullivan G., Kaplan R. (2003). Comparing the sensitivity of generic effectiveness measures with symptom improvement in persons with schizophrenia. Med Care.

[28] Russo J., Trujillo C.A., Wingerson D. (1998). The MOS 36-item short form health survey: reliability, validity, and preliminary findings in schizophrenic outpatients. Med Care.

[29] Sciolla A., Patterson T.L., Wetherell J.L. (2003). Functioning and well-being of middle-aged and older patients with schizophrenia: measurement with the 36-item short-form (SF-36) health survey. Am J Geriatric Psych.

[30] Konig H.H., Roick C., Angermeyer M.C. (2007). Validity of the EQ-5D in assessing and valuing health status in patients with schizophrenic, schizotypal or delusional disorders. Eur Psychiatry.

[31] Konig H.H., Gunther O.H., Angermeyer M.C. (2009). Utility assessment in patients with mental disorders: validity and discriminative ability of the time trade-off method. Pharmacoeconomics.

[32] Pukrop R., Schlaak V., Moller-Leimkuhler A.M. (2003). Reliability and validity of quality of life assessed by the short-form 36 and the modular system for quality of life in patients with schizophrenia and patients with depression. Psych Res.

[33] Kebede D., Alem A., Shibre T. (2004). Health related quality of life (SF-36) survey in Butajira, rural Ethiopia: normative data and evaluation of reliability and validity. Ethiop Med J.

[34] Kebede D., Alem A., Shibre T. (2005). Short-term symptomatic and functional outcomes of schizophrenia in Butajira. Ethiop Med J.

[35] Auquier P., Simeoni M.C., Sapin C. (2003). Development and validation of a patient-based health-related quality of life questionnaire in schizophrenia: the S-QoL. Schizophren Res.

[36] Reine G., Simeoni M.C., Auquier P. (2005). Assessing health-related quality of life in patients suffering from schizophrenia: a comparison of instruments. Eur Psychiatry.

[37] Bobes J., Gonzalez M.P., Saiz P.A. (1997). The SF-36 versus the WHOQOL-100 and -26 in schizophrenic private out-patients. Qual Life Res.

[38] Prieto L., Sacristan J.A., Hormaechea J.A. (2004). Psychometric validation of a generic health-related quality of life measure (EQ-5D) in a sample of schizophrenic patients. Curr Med Res Opin.

[39] Meijer C.J., Schene A.H., Koeter M.W. (2002). Quality of life in schizophrenia measured by the MOS SF-36 and the Lancashire quality of life profile: a comparison. Acta Psychiatrica Scandinavica.

[40] van de Willige G., Wiersma D., Nienhuis F.J., Jenner J.A. (2005). Changes in quality of life in chronic psychiatric patients: A comparison between EuroQol (EQ-5D) and WHOQoL. Qual Life Res.

[41] Barton G.R., Hodgekins J., Mugford M. (2009). Measuring the benefits of treatment for psychosis: validity and responsiveness of the EQ-5D. Br J Psychiatry.

[42] Wilkinson G., Hesdon B., Wild D. (2000). Self-report quality of life measure for people with schizophrenia: the SQLS. Br J Psychiatry.

[43] Sanderson K., Andrews G., Sanderson K., Andrews G. (2002). Prevalence and severity of mental health-related disability and relationship to diagnosis. Psychiatric Serv.

[44] Milliken H.I., Whitehorn D., Rui Q., Ronson K. (2007). SF-36: a measure of recovery in the first six months of treatment for schizophrenia. Schizophr Bull.

[45] Norholm V., Bech P., Norholm V., Bech P. (2007). Quality of life assessment in schizophrenia: applicability of the Lehman quality of life questionnaire (TL-30). Nord J Psychiatry.

[46] Law C.W., Chen E.Y., Cheung E.F. (2005). Impact of untreated psychosis on quality of life in patients with first-episode schizophrenia. Qual Life Res.

[47] Scalone L., Pirfo E., Mencacci C. (2008). Relationship between clinical outcomes and patients' reported outcomes in schizophrenia: the contribution of the EQ-5D. Value Health.

[48] Jarema M., Konieczynska Z. (2001). Quality of life in schizophrenia: Impact of psychopathology, patients' gender and antipsychotic treatment. Int J Psychiatry Clin Pract.

[49] Badia X, Casado A, Sacristan JA, et al. Antipsychotic treatment and changes in health related quality of life in patients with schizophrenia using EQ-5D. Abstract from the Second Annual European Meeting of the International Society for PharmacoEconomics and Outcomes Research, Edinburgh, Scotland, November 1999.

[50] Dunayevich E., Ascher-Svanum H., Zhao F. (2007). Longer time to antipsychotic treatment discontinuation for any cause is associated with better functional outcomes for patients with schizophrenia, schizophreniform disorder, or schizoaffective disorder. J Clin Psych.

[51] U.S. Department of Health and Human Services Guidance for industry: patient-reported outcome measures: use in medical product development to support labeling claims. http://www.fda.gov/downloads/Drugs/GuidanceComplianceRegulatoryInformation/Guidances/UCM193282.pdf.

[52] European Medicines Agency Pre-authorisation Evaluation of Medicines for Human Use: Reflection paper on the regulatory guidance for the use of health-related quality of life (HRQL) measures in the evaluation of medicinal products. http://www.ema.europa.eu/docs/en_GB/document_library/Scientific_guideline/2009/09/WC500003637.pdf.

[53] Brazier J., Tsuchiya A. (2010). Preference-based condition-specific measures of health: what happens to cross programme comparability?. Health Econ.

