# Supplementary material for: How Valid and Responsive Are Generic Health Status Measures, such as EQ-5D and SF-36, in Schizophrenia? A Systematic Review
Source: Value Health. 2011 Sep;14(6):907–20. doi: 10.1016/j.jval.2011.04.006 (PMC3179985; doi:10.1016/j.jval.2011.04.006)
Supplement: Appendix [file mmc1.doc]

Appendix: SF-36 validity and responsiveness

|  | | **Measurement and Practical Properties** | | |
| --- | --- | --- | --- | --- |
| **SF-36** | | | | |
| **Study details** | **Population characteristics** | **Properties measured** | **Source and types of measures used to test convergent validity and/or responsiveness*** | **Details of** **validity or responsiveness** |
| Auquier, P (2003)(36)  France | DSM-IV schizophrenia  Inpatients and outpatients (numbers not reported)  N=207  141 males and 68 females  Mean age= 37.3 (SD=10.9)  Age range= 18 to 70 years | Convergent validity | **Patient-completed** | Significant and strong/very strong correlations between S-QoL domains and related SF-36 domains. For example: S-QoL Psychological well-being and SF-36 social functioning ( 0.58, p<0.05) and mental health (0.65, p<0.05); S-QoL self-esteem and SF-36 mental health (0.60, p<0.05); S-QoL physical well-being and SF-36 vitality (0.56, p<0.05).  Mostly weaker correlations between three S-QoL subscales that were not covered by the SF-36: autonomy (0.29 to 0.40), resilience (0.17 to 0.36) and sentimental life (0.13 to 0.40).  Contradictory evidence: weak correlation between S-QoL subscales family relationships and relationships with friends and the SF-36 social functioning domain (0.18 and 0.26 respectively). |
|  |  |  | 1. i) Quality of life- disease specific   S-QOL |  |
| Bebbington, PE (2009)(21)  UK, France, Germany | DSM-IV schizophrenia  N= 1208  743 males and 465 females.  Aged between 18 and 64. No mean age reported.  Outpatients  All on antipsychotic medication (type not specified) | Known groups validity | Not applicable | Significant differences in MCS and PCS between participants with or without side effects from antipsychotic medication.  Some side effects were not detected on the SF-36 and may need to be assessed separately in terms of QoL effect |
| Bobes, J (1997)(38)  Spain | ICD-10 schizophrenia  All outpatients- measured by PANSS as out of acute phase; on maintenance treatment with different neuroleptics  N= 60 patients  No age or gender data reported. | Convergent validity | **Patient-completed** | Most but not all SF-36 domains correlated significantly with two versions of the WHO-QOL: WHOQOL-11 and WHOQOL-26  Ranged from 0.2583 (psychological domain on WHOQOL-100 and SF-36 vitality scale) to 0.5774 (between overall WHOQoL-100 and social functioning).  WHOQOL-26 correlated significantly with general health, social functioning, role emotional and mental health (ranged from 0.2599 to 0.6008). No correlation with physical functioning, role physical, bodily pain and vitality scales. |
|  |  |  | 1. Quality of life-generic   WHO-QOL |  |
| Dunayevich, E (2007)(51)  Unknown | DSM-IV schizophrenia (n=1278, 78.5%), schizoaffective disorder (n=339, 20.8%) or schizophreniform disorder (n=10, 0.6%)  Inpatient/outpatient stauts not reported  Pools results from four studies:  **Study 1:** N=339, 220 males and 119 females; Mean age= 36.2 (SD=10.7)  Olanzapine versus risperiodone  **Study 2 :** N=346, 228 males and 118 females; Mean age=41.1 (SD=9.6)  Olanzapine versus quetiapine  **Study 3:** N=548, 352 males and 196 females; Mean age= 39.2 (SD=11.9)  Olanzapine versus ziprasidone  Study 4: N=394, 248 males and 146 females; Mean age=39.5 (SD=10.9)  Olanzapine versus ziprasidone | Responsiveness | **Clinician-completed** | Weak to moderate correlations with time to treatment discontinuation (used by the authors as a proxy measure for efficacy, safety and tolerability) and the SF-36 domains and the two summary scores.  Weak correlations between symptom improvement on the PANSS (total score and PANSS domains) and functional improvement on the SF-36 MCS (range 0.241 to 0.341).  Effect sizes calculated using scores for treatment completers and non-completers at week 8, 16 and 24 after study baseline were mostly weak to moderate on the MCS 0.39 to 0.53) and weak on the PCS (0.11 to 0.34). In general, effect sizes were stronger on the MCS domains of vitality, social functioning, role emotional and mental health than on PCS domains. |
|  |  |  | 1. Symptoms   PANSS |  |
| Folsom, D (2009)(25)  USA | DSM-IV schizophrenia or schizoaffective disorder  N=164 (42.3%) on atypical antipsychotics  n=198 (51.1%) on atypical antipsychotics  Outpatients  N=486  317 males and 169 females  Mean age=54.7 (SD=8.6)  No age range given | Known groups validity | Not applicable | Individuals with schizophrenia and schizoaffective disorder had significantly lower scores on the PCS (3.6 points, p=0.002) and MCS (10.4 points, p<0.001) than normal comparison participants |
| Jarema M (2001)(49)  Poland | DSM-IV schizophrenia  Comparison of classic versus atypical neuroleptics- patients were admitted with a ‘worsening mental state’. [Baseline and end of study]  Inpatients  N=199  40 males and 60 females  Mean age= 41.5 (SD=9.6)  Age range=19 to 66 years | Convergent validity  Responsiveness | **Clinician-completed** | Moderate correlations with SF-36 total score (study does not report MCS or PCS scores, and does not state how total SF-36 calculated) and PANSS depressive symptoms domain (0.318 at baseline and 0.397 at end of study, both p=0.001). Moderate SF-36 total score with PANSS total score at the end of the study (0.315, p=0.001). Some MCS domains) were significantly but weakly/moderately correlated (0.206 and 0.419) with PANSS total score and PANSS depressive symptoms.  Weak to moderate correlations with changes scores on symptoms measures and SF-36 dimensions were only significant between the PANSS general and depressive subscales and the SF-36 total scores, mental health and vitality domains (range 0.211 to 0.361). |
|  |  |  | 1. Symptoms   PANSS |  |
| Kebede, D (2004)(34)  Ethiopia | ICD-10 schizophrenia  N=307  253 males and 54 females  Mean age and age range not reported.  Outpatients | Known groups validity | Not applicable | Considerable differences between SF-36 scores of individuals with schizophrenia in comparison with normative values. MCS domains approximately 20 to 80 points lower and PCS domains approximately 25 to 40 points lower for individuals with schizophrenia. No tests of statistical significance undertaken. |
| Kebede, D (2005)(35)  Ethiopia | ICD-10 schizophrenia  N= 271/321 available for follow-up analysis (4 years)  220 males and 51 females  208 defined as being long-standing cases (duration of illness between 3 and 30 years) and 63 defined as having recent onset illness (onset within the last 2 years).  Mean age=30.8 (0.5)  Age range not given  Outpatients | Known groups validity  Responsiveness | **Clinician-completed** | At baseline and follow-up periods of 1, 2, 3 and 4 years, mean SF-36 dimension scores were lower than the mean scores of the general population in Butajira, Ethiopia for both long-standing and recent-onset schizophrenia. This was statistically significant (p<0.01) for all dimensions. PCS scores ranged approximately between 25 to 50 points lower, whereas MCS scores ranged between 20 to 80 points lower.  SANS and SAPS scores were inversely related with improvements in physical and social functioning domains (p=0.001) and role limitations due to emotional problems (p<0.05). |
|  |  |  | 1. Symptoms   SAPS, SANS |  |
| Law, C (2005)(47)  Hong Kong | ICD-10 schizophrenia  Untreated psychosis- participants experiencing first episode  Inpatients and outpatients (mostly outpatients, numbers not reported)  N=117  63 males and 54 females  Mean age=20.3 years (SD=3.6)  Range= 14 to 28 years | Known groups validity  Convergent validity | **Clinician-completed** | Young people with schizophrenia had significantly lower SF-36 scores on all domains than general population (p<0.001) except on role emotional (p=0.003). PCS domain scores were approximately 10-40 points lower and MCS were approximately 10-30 points lower in individuals with schizophrenia  SF-36 domains had no significant correlations with symptoms measures (SANS and PANSS). Weak correlations with the MADRS (range 0.12 to 0.27) and only three were statistically significant (p<0.05): role physical, general health and vitality.  WHOQOL-BREF correlated significantly with both PCS and MCS. Strongest correlations seen between WHO-QOL Psychological Health domain and mental health domain, 0.69, p<0.01. |
|  |  |  | 1. Symptoms   PANSS, MADRS, SANS |  |
|  |  |  | **Patient-completed** |  |
|  |  |  | 1. Quality of life- generic   WHOQOL-BREF |  |
| Lenert, L (2005)(14)  Europe and Canada | DSM-IV schizophrenia or schizoaffective disorder  N=725 completed the trial.  N=615 schizophrenia N=110  312 males and 164 females completed the trial  Treatment: all on risperidone of varying doses  Mean age for completers=43.7 (SD=0.7)  No age range given  Inpatients/outpatients status not reported | Convergent validity | **Clinician-completed** | The SF-36 mapping function was significantly correlated to PANSS utility values but only weakly (r=0.2) |
|  |  |  | 1. Symptoms   PANSS |  |
| Meijer, C (2002)(40)  Netherlands | ICD-10 schizophrenia/SCAN diagnosed schizophrenia  N=143  Aged 18-65 years  No mean age or gender data reported  Inpatients and outpatients (numbers not reported) | Convergent validity | **Clinician-completed** | Weak to moderate correlation with the GAF functioning measures ranging between 0.17 and 0.38. Strongest correlation on social functioning (0.37) and mental health domains (0.38). LQoLP did not perform better. |
|  |  |  | 1. Functional   GAF   1. Quality of life- disease-specific   LQoLP |  |
| Milliken, H (2007)(45)  Canada | Schizophrenia spectrum disorder (classification not specified)-  Treatment < 6 months exposure to antipsychotic medication  Inpatients/outpatients status not reported  N=15 (first patients with complete SF-36 data)  No age or gender data reported. | Convergent validity  Responsiveness | **Clinician-completed** | Strong correlations with symptoms (PANSS, 0.861, p=0.001)) and functioning measures (GAF, 0.722, p=0.012 and SOFAS, 0.899, p=0.001). There was a trend for higher MCS scores for remitted compared to non-remitted patients (p=0.063) |
|  |  |  | 1. Symptoms   PANSS   1. Functioning   GAF, SOFAS |  |
| Nasrallah, H (2004)(26)  USA | DSM-IV schizophrenia  Inpatients or outpatients (numbers not reported)  Treatment: Long-acting injection and oral risperidone vs. oral risperidone  N= 369  Mean age across four treatment groups ranged between 36.0 (SD=1.0) and 39.0 years (SD=1.1).  275 males and 94 females | Known groups validity | Not applicable | Lower scores on all domains expect body pain for individuals with schizophrenia compared with general US population (p<0.05). Largest differences were seen in the mental health domain (approximately 28 points), role emotional (8.5) and vitality (9.34) |
| Norholm, V (2007)(46)  Denmark | ICD-10 schizophrenia  Inpatients- during hospitalisation  Usual treatment including antipsychotic medication, psychosocial support and a rehabilitation programme  N=85  23 males and 33 females  Mean age= 39 (SD=10.9)  Range 21-65 years  Compared with an age-matched sample of Danish population (N=2650) | Known groups validity | Not applicable | Significant differences on all SF-36 domains except bodily pain when inpatients and normative data from Danish population compared (p≤0.01) (Difference in points not reported). |
| Phillips, G (2006)(27)  USA | DSM-IV Schizophrenia  Treatment; planzapine vs. ziprasidone  N=548  n=277 assigned to olanzapine and n=271 ziprasidone  Mean age in olanzapine group= 40.1 and mean age in ziprasidone group =38.2  No gender data reported  Inpatients and outpatients (numbers not reported) | Responsiveness | **Clinician-completed** | Mostly weak to moderate correlations between changes on QLS and changes on SF-36 (p<0.05) (Exception= SF-36 bodily pain and QLS interpersonal relationship). Strongest correlation between SF-36 mental health and the QLS intrapsychic foundations dimension (r=0.408, p<0.0001).  Small to moderate correlations between SF-36 change scores and changes on PANSS total (0.216 to 0.381), PANSS positive (0.198 to 0.319) and PANSS negative (0.159 to 0.276). All p<0.001.  Generally correlations stronger on MCS (0.299 to 0.381) than PCS (0.181 to 0.224). |
|  |  |  | i) Symptoms  PANSS  ii) Quality of life-disease specific  QLS (interviewer administered) |  |
| Pukrop, R (2003)(33)  Germany | DSM-IV schizophrenia  Inpatients  N=91  47 males and 44 females  Mean age= 33.73 (SD=9.67) | Known groups validity  Convergent validity  Responsiveness | **Clinician-completed** | Significant differences (mostly p<0.001) in SF-36 scores compared with healthy controls, at admission (range11.7 to 47.0) and discharge (6.2 to 38.1).  Only one statistically significant and correlation with the PANSS score and social functioning domain which was moderate (0.36).  Significant improvement in all SF-36 dimensions from admission to discharge. SF-36 improvements showed no interaction with improvement in positive symptoms except for physical functioning and bodily pain. These became insignificant when controlling for improvement in positive symptoms. Improvement in negative symptoms significantly impacted on role physical and role emotional dimensions. When improvement in negative symptoms was controlled for, all SF-36 improvement remained significant. |
|  |  |  | 1. Symptoms   PANSS, HAM-D |  |
| Pyne, J (2003)(28)  USA | DSM-IV schizophrenia or schizoaffective disorder  N=134 (n=85 schizophrenia; n=49 schizoaffective disorder)  Inpatients and outpatients (numbers not reported)  126 males and 8 females  Mean age=46.8 (SD=8.1) (No range reported) | Responsiveness | **Clinician-completed** | Clinically significant symptom improvement was defined by a 20% improvement in total PANSS score. Correlation with change scores on the PANSS were very weak, ranging 0.06 to 0.18 on the SF-36 VAS and 0.009 and 0.12 on the SF-36 MCS.  Correlations with the SF-36 MCS depression measure (CDSS, 0.27) and a side effects measure (ESRS, 0.22) were also weak. The effect size for the MCS was very small at 0.16 |
|  |  |  | 1. Symptoms   PANSS, CDSS, ESRS |  |
| Reine, G (2005)(37)  France | DSM-IV schizophrenia  36% outpatients & 64% inpatients  Phase: first episode (7%); readmission period for an acute phase (31%) and stabilised (62%)  N=205 (139 males, 66 females)  Mean age=37.4 (SD=10.9) [Age range=18-70 years] | Convergent validity  Responsiveness | **Clinician-completed** | Correlations with PANSS Total and PANSS subscales, CGI, GAF and ESRS were weak on both the MCS and PCS and individual SF-36 domains (all <0.3) but statistically significant. Some correlations with the CDSS were moderate (MCS summary scores and individual MCS domains).  Small effect sizes recorded (0.01 to 0.41) between the SF-36 scores on the patients judged to have improved between the two visits within the study (based on CGI-S score). None were significant apart from SF (0.41, p<0.05) and RP (0.40, p<0.05). |
|  |  |  | 1. Symptoms   PANSS, ESRS, CGI-S &  CDSS   1. Functional   GAF |  |
| Revicki, D A (1999)(22)  Austria, Belgium, France, Germany, Italy, Poland, Spain, USA, Canada, UK | DSM-III-R Schizophrenia, schizophreniform disorder and schizoaffective disorder  Inpatients or outpatients (numbers not reported)  BPRS score ≤18  Treatment: olanzapine vs. haloperidol RCT  N=1159 (787 olanzapine patients and 372 haloperidol patients)  Age and gender not reported for this group | Convergent validity  Responsiveness | **Clinician-completed** | Weak correlation with a disease-specific quality of life measure (QLS) ranging from 0.03 to 0.25.  A regression model for baseline to endpoint change in total MCS Scores resulted in an R^2^ of 0.25 and showed statistically significant contributions for changes in PANSS positive scores (p<0.001) and for changes in MADRS scores (p<0.0001). i.e. Decreases in positive symptoms (e.g. hallucinations, delusions, disrupted thought) and depression symptoms were associated with increases in psychological well-being (MCS) |
|  |  |  | 1. Symptoms   PANSS, MADRS  ii) Quality of life-disease  specific  QLS (interviewer administered) |  |
| Russo, J (1998)(29)  USA | DSM-III-R schizophrenia or schizoaffective disorder  Outpatients  N=36  25 males and 11 females  Mean age: 40.0 (SD=8.07)  Range= 19 to 53 | Known groups validity  Convergent validity | **Clinician-completed** | Mean scores of MCS and PCS dimensions were significantly lower in individuals with schizophrenia when compared with population values. MCS dimension were approximately 10 to 40 points lower and PCS scores 5 to 25 points lower.  Total average BPRS score was significantly related to the subscales role emotional (r= 0.57, p<0.001); and social functioning(r=0.60, p<0.001). Associations were also present between the BPRS and mental health (r=0.43, p<0.01) and vitality (r=0.36, p<0.05) and the role physical (r=0.44, p<0.01).  Similarly moderate to strong significant (p<0.01) correlations were found between the BPRS depression and anxiety subscales and role emotional, social functioning, mental health |
|  |  |  | 1. Symptoms   BPRS |  |
| Scalone, L (2008)(48)  Italy | N= 637 (n=551with schizophrenia  n=86 with schizophreniform disorder )  414 males and 223 females  Aged 18 to 40 years (no mean age reported)  Inpatients/outpatients status not reported | Convergent validity | **Clinician-completed** | Weak to moderate correlations between QoL scores (EQ-5D and SF-36) and symptom measures (PANSS and CGI-S) ranging from 0.189 to 0.393. |
|  |  |  | 1. Symptoms   PANSS, CGI-S |  |
| Sciolla,A (2003)(30)  USA | DSM-IV schizophrenia or schizoaffective disorder  ‘Medically, psychological and pharmacologically stable’  Mostly outpatients (numbers not reported)  N=137  91 males and 46 females  Mean age= 57.9 (8.9)  N=77 ‘normal’ participants  30 males and 47 females  Mean age= 66.0 (10.6) | Known groups validity | Not applicable | Mean scores on all SF-36 subscales and composite scores were significantly lower (p value ranged <0.001 and <0.05) for individuals with schizophrenia (or schizoaffective disorder) than ‘normal’ controls with the exception of bodily pain (p=0.37). Largest differences seen on the mental health, role emotional and social functioning scale (approximately 17 points difference). . |
| Strakowski, S M (2005)(23)  USA and Western Europe | DSM-IV Schizophrenia, schizoaffective disorder and schizophreniform disorder  First episode of psychosis- active psychotic symptoms (at least two PANSS psychosis items ≥4 or one psychosis item ≥5, and a CGI score ≥4  Inpatients/outpatients status not reported  RCT olanzapine vs. haloperidol  N= 195  156 males and 39 females  Mean age= 24 years (SD=5)  No range reported  Normative data were taken from published tables (Ware et al 1994) and were weighted to the age and gender of the study sample | Known groups validity | Not applicable | Baseline scores were significantly below normative scores for all SF-36 subscales except physical functioning. MCS domains were 13 to 50 points lower in individuals with schizophrenia when compared with normative scores (highest on mental health domain). PCS domains were 3 to 19 points lower than normative scores. |
| Tunis, S L (1999)(24)  USA, UK and Canada | DSM-III-R schizophrenia, schizophreniform disorder or schizoaffective disorder  Experiencing ‘clinically significant psychosis’  Inpatients and outpatients (numbers not reported)  N=1,155  802 males and 353 females  Mean age= 39.29 (SD=11.32)  Age range not reported.  n=935 schizophrenia and n=220 schizoaffective disorder. | Known groups validity  Convergent validity | **Clinician-completed** | Significant differences (p<0.001) in scores between individuals with schizophrenia and general population on all domains except bodily pain. Larger differences were seen on MCS domains (13-40) than PCS domains (1-14)  Weak to moderate significant (p<0.001) correlations between the MCS, general health, vitality, social functioning, role emotional and mental health domains with BPRS (0.20 to 0.316). Weak significant (p<0.001) correlations with MCS and social functioning and CGI-S (0.15 and 0.20 respectively). Mostly moderate to strong significant (p<0.001) correlations with MADRS and (0.20 to 0.58) |
|  |  |  | 1. Symptoms   BPRS, MADRS, CGI-S |  |
| Wilkinson, G (2000)(43)  UK | Schizophrenia (unknown classification)  Clinic and home-based settings  N=78 (male/female not reported)  Mean age=40(11.9)  Age range= 18-64  Inpatients/outpatients status not reported. | Convergent validity | **Patient-completed** | Predicted correlations between SQLS and SF-36 were demonstrated and were very strong and statistically significant. SF-36 Vitality/SQLS ‘motivation and energy’: 0.72, p<0.001, SF-36 MH/SQLS psychosocial= 0.65, p<0.001 |
|  |  |  | 1. Quality of life- disease specific   SQLS |  |

* Note other measures used in the study but not used to test convergent validity or responsiveness are not listed

BPRS=Brief Psychiatry Rating Scale; CDSS= Calgary Depression Scale for Schizophrenia, CGI-S= Clinical Global Impression-Severity , ESRS= Extrapyramidal Symptom Rating Scale; GAF=Global Assessment of Functioning; HAM-D= Hamilton Depression Rating Scale; LQolP= Lancashire Quality of Life Profile; MADRS= Montgomery-Åsberg Depression Rating Scale; PANSS= Positive and Negative Syndrome Scale ; QLS= Quality of Life Scale; QoLI= Quality of Life Inventory; QWB= Quality of Well-Being; SANS= Scale for the assessment of négative symptoms ; SAPS= Scale for the assessment ofpositive symptoms ; SOFAS= Social and Occupational Functioning Assessment Scale ; SQLS= Schizophrenia Quality of Life Scale; S-QOL= Schizophrenia Quality of Life questionnaire ; WHO-QOL= WHO Quality of Life
